# The Potential Benefits and Controversies of Probiotics Use in Patients at Different Stages of Chronic Kidney Disease

**DOI:** 10.3390/nu14194044

**Published:** 2022-09-29

**Authors:** Na Tian, Lu Li, Jack Kit-Chung Ng, Philip Kam-Tao Li

**Affiliations:** 1 Department of Nephrology, General Hospital of Ningxia Medical University, Yinchuan 750004, China; 2 Department of Medicine and Therapeutics, Prince of Wales Hospital, The Chinese University of Hong Kong, Ngan Shing St., Shatin, Hong Kong, China; 3 Carol and Richard Yu Peritoneal Dialysis Research Centre, The Chinese University of Hong Kong, Ngan Shing St., Shatin, Hong Kong, China

**Keywords:** probiotics, chronic kidney disease, dialysis, gut dysbiosis

## Abstract

The therapeutic modulation of the gut microbiome has been suggested to be one of the tools in the integrated management of chronic kidney disease (CKD) in recent years. Lactobacillus and Bifidobacterium genera are the two most commonly used probiotics strains. Most of the probiotics used in studies are mixed formulation. There is no consensus on the dose and duration of the probiotic administration for CKD patients Increasing evidence indicates that patients with early stage (1–2) CKD have an altered quantitative and qualitative microbiota profile. However, there was a dearth of prospective controlled studies on the use of probiotics in the early stage of the CKD population. The association between gut microbiota disturbance and advanced CKD was reported. Most randomized controlled trials on probiotic treatment used in CKD stage 3–5ND patients reported positive results. The metabolites of abnormal gut microbiota are directly involved in the pathogenetic mechanisms of cardiovascular disease and inflammation. We summarized 13 studies performed in the dialysis population, including 10 in hemodialysis (HD) patients and 3 in peritoneal dialysis (PD). Some controversial results were concluded on the decreasing plasma concentration of uremic toxin, symptoms, inflammation, and cardiovascular risk. Only three randomized controlled trials on PD were reported to show the potential beneficial effects of probiotics on inflammation, uremic toxins and gastrointestinal symptoms. There is still no standard in the dosage and duration of the use of probiotics in CKD patients. Overall, the probiotic administration may have potential benefit in improving symptoms and quality of life, reducing inflammation, and delaying the progression of kidney failure. Further research studies using a larger sample size with longer follow-up durations and a greater focus on clinical outcomes—including survival—are warranted to elucidate the significant clinical impact of the use of probiotics in CKD patients.

## 1. Introduction 

Gut microbiota is involved in metabolic homeostasis, as shown in studies with humans. In recent decades, there is increasing evidence of the dysbiotic microbiota in the chronic kidney disease (CKD) population. Crosstalk does exist between the intestine and the kidney, which is the so-called kidney–gut axis. The gut microbiota interacts with the kidneys by very complicated mechanisms, including diet, microbiota-derived uremic toxins, immune-mediated factors and metabolites, such as short-chain fatty acids (SCFAs). The overgrowth of the proteolytic bacteria (actinobacteria, proteobacteria, and firmicutes) was promoted by urea [1], by way of inducing the translocation of bacteria or their fractions into the bloodstream, and increasing the permeability of the intestinal wall, which in turn may enhance accelerated atherosclerosis and systemic inflammation [2].

With the progression of renal dysfunction, the accumulation of uremic toxins may further amplify their deleterious effects. The diversity and quantity of bacteria and the production of uremic toxins, including p-cresyl sulfate (PCS) and indoxyl sulfate (IS), varied with different stages of kidney function [3] and different dialysis modalities [4]. The therapeutic modulation of the gut microbiome was suggested to be one of the tools in the integrated management of CKD in recent years, with a view to delay the deterioration of kidney function, prevent and treat CKD-related complications, and improve the gut microenvironment. Probiotics, prebiotics, and synbiotics are the most commonly used therapeutic agents to modulate gut dysbiosis.

This review aimed to focus and evaluate the effects of probiotics for patients with early CKD stage, advanced CKD stage and on maintenance dialysis therapy. All the literature was searched in Pubmed (English database) using keywords (gut dysbiosis AND chronic kidney disease; probiotics AND chronic kidney disease; dysbiosis AND hemodialysis; probiotics AND hemodialysis; dysbiosis AND peritoneal dialysis; probiotics AND peritoneal dialysis), with publication years from 2000 to 2022.

## 2. Overview of Probiotics Used in CKD

### 2.1. Type, Dose, and Intervention Duration of Probiotics 

The International Scientific Association for Probiotics and Prebiotics (ISAPP) consensus defines probiotics as “live microorganisms which when administered in adequate amounts confer a health benefit on the host” [5]. Probiotics are live and vital microorganisms which may be beneficial as part of a food or a supplement. The most frequently administered probiotics are *Bifidobacteria longum*, *B. bifidum*, *Lactobacillus acidophilus*, *L. casei*, *L. sakei*, *L. reuteri*, and *Streptococcus thermophilus*. Bifidobacteria are capable of producing vitamins, such as vitamin B1, B4, B6, B12, folate, and nicotinic acid [6]. Evidence from both in vivo and in vitro models has shown that the Bifidobacteria could effectively ameliorate epithelial damage, restore epithelial function [7,8], and produce short-chain fatty acids, particularly butyrate, through a cross-feeding mechanism by stimulating the growth of other bacterial species such as *Lactobacillales* [7]. *L. acidophilus* and *B. bifidum* have immune enhancement and stabilization effects on the gut mucosal barrier [9]. Enterococcus faecalis can generate bacteriocins (antimicrobial peptides) [10], which can inhibit the overgrowth of pathogens in the colon of CKD patients. 

We summarized 25 original research studies which examined the benefit of probiotics in CKD patients. We included 24 randomized controlled studies and one cross-sectional study, which were conducted in CKD 3–5 non-dialysis stage as well as in dialysis patients. Lactobacillus and Bifidobacterium genera were the two most commonly used probiotics strains. In these studies, most of the microorganisms belonged to the Lactobacillus (19 studies, 73%) and Bifidobacterium genera (17 studies, 65%). Most of the probiotics used in the studies were mixed formulation, accounting for 80% of the total. Multiple probiotic products are now marketed and the efficacy, safety, and tolerance were shown in some trials [11,12,13].

The exact dose of probiotic that should be administered to CKD patients and for how long remain unclear. Insufficient doses of probiotics can be a potential factor leading to insignificant effects. The choice of the strain of probiotic used has until now been mainly empirical, contributing to the discrepancies between studies. Interventions usually last several weeks, but could last between 4 weeks and 5 years. Similarly, the probiotic dosages ranged from 16 × 10^9^ CFU to 2.0 × 10^12^ CFU; the dosages strategy had no specific standard. The formulation of the probiotic agents varied; some were administered in capsules, some were given in bags/envelopes to be dissolved in water or milk, while others were added to yogurt. The administration times of the probiotics reported in the studies included with meals or right after meals.

Another interesting issue is whether the living microorganisms could “safely” arrive the intestines and become colonized. Taki et al. found that the Bifidobacteria in most medicinal products and health foods cannot usually survive at pH 1.2 when it was exposed to gastric juices through oral administration. A gastro-resistant seamless capsule protects acid-fragile *Bifidobacterium longum* from the acidic gastric juice until they reach the intestines. The capsule protects Bifidobacteria from inactivation by acidic gastric juice, preserving its activity in the intestines [14].

### 2.2. Dietary Interventions other Than Probiotics

The colon-derived solutes included PCS and IS, which are produced by the bacterial metabolism of the amino acids tyrosine and tryptophan, respectively. The alteration of diet can influence the production of colon-derived solutes. In addition to probiotics, prebiotics, and synbiotics, the therapeutic interventions for gut flora regulation include the dietary use of fruits, vegetables, and high-fiber products. The Atherosclerosis Risk in Communities (ARIC) study [15] included 12,000 adults with normal kidney function. The use of a healthy diet including many fruits and vegetables, fish, legumes, whole grains, and fibers with decreased red meat, sodium, and refined sugar intake were associated with a reduced risk of incident CKD for those subjects consuming more vegetable proteins and conferred a lower mortality in CKD patients [15,16]. Another study showed that the red meat intake was associated with a higher risk of developing end-stage kidney disease [17]. In the general population with normal kidney function, a vegetarian diet reduces the urinary excretion of PCS and IS by approximately 60% [18]. However, a diet with a higher content of non-digestible fibers, as consumed by vegetarians, may explain the prebiotic effect modulating uremic solutes production rather than the reduction in animal proteins [19]. In other countries, several examples of traditional food made by fermentation process contained large quantity of probiotics were taken as a daily food by healthy people. Wagner et al. [20] studied 888 CKD stage 3–5 patients and noted that yoghurts and probiotics (irrespective of the frequency of intake) are associated with reduced inflammation. Compared to subjects not consuming yoghurt, the ORs [95% CI] for CRP > 6 or >7 mg/L were significantly lower for those consuming ordinary yoghurt (0.58 [0.37, 0.93] and 0.57 [0.35, 0.91], respectively) and for those consuming probiotics (0.54 [0.33, 0.9] and 0.48 [0.28, 0.81], respectively). However, no dose–effect relationship was observed [20]. 

## 3. Effect of Probiotics in Early Stage of CKD (Stage 1–2)

In patients in early stages of CKD, the quantitative and qualitative profile of microbiota might be changed [1,21]. There was marked differentiation in the levels of metabolites (free amino acids and organic volatile compounds) from fecal and urinary samples between the progressor and non-progressor of patients with IgA nephropathy [22]. In CKD, the levels of both urea and ammonium increase in the gut, raising the pH level and promoting aerobic bacteria growth. In turn, these aerobic bacteria produce uremic toxins such as PCS, IS, and trimethylamine N-oxide which decrease the number of healthy anaerobic bacteria in the gut [23]. There was a dearth of prospective controlled studies on the use of probiotics in the early stage (1–2) of the CKD population. A case–control study revealed that the bacteria were not related to triglyceride, cholesterol, BUN, and creatinine levels; however, a negative correlation between Roseburia spp., Faecalibacterium prausnitzii, and CRP and renal function suggested that the depletion of butyrate-producing bacteria may contribute to CKD-associated inflammation and CKD progression [24]. In a randomized control trial, soy milk with *Lactobacillus plantarum* A7 was found to significantly reduce the oxidized glutathione concentration when it had been administrated to diabetes patients with proteinuria > 300 mg/day and glomerular filtration rate > 90 mL/minute for 8 weeks [25].

## 4. Effect of Probiotics in Advanced Stage of CKD (Stage 3–5ND)

Patients with advanced CKD are commonly recommended that to restrict their vegetable and fruit intake to reduce the danger of hyperkalemia and fluid overload. The shortage of fiber predisposes to dysbiosis with intestinal transit slowing, intestinal wall edema, and metabolic acidosis. Furthermore, polypharmacy, which is common in patients with end-stage kidney disease (ESKD) (including the use of vitamin D analogs, potassium, iron, phosphate-lowering agents, and diuretics), induced pro-inflammatory gastrointestinal overload [21,26]. Fermentative dysbiosis may be attributed to non-absorbed sugar hydrolyzation by several bacteria strains in the ascending colon and in the caecum. 

To date, most randomized controlled trials on probiotic treatment used in the CKD stage 3–5ND patients have reported positive results (Table 1). The colon-derived uremic toxin levels, such as PCS, IS, phenylacetylglutamine, and serum trimethylamine N-oxide, were commonly studied and a significant reduction in these uremic toxins by probiotics were shown in patients entering an advanced stage of CKD [23,27,28,29,30]. Guida et al. reported the effect of Probinul neutro^®^ on 30 patients in stage 3–4 CKD in a randomized control trial for 4 weeks. They observed a significant decrease in the total plasma p-cresol levels however, without any improvement in GI symptoms [31]. The SYNERGY trial, with 37 stage 4–5ND CKD patients, demonstrated a decrease in serum p-cresyl sulfate, however, not in indoxyl-sulfate and a favorable change in the stool microbiome [29]. A significant change in both gut microbiota composition and intestinal bacterial metabolism was found in most of patients after taking Lactobacillales and Bifidobacteria [32,33]. The fecal pH measured in a multi-centered pilot study showed that the probiotic bacteria cohort (pH 6.94) was much lower than the placebo cohort (pH 7.29), which could be partially explained by the production or generation of lactic acid Lactobacillus in the mixture administered to the probiotic cohort [30]. 

Given the close relationship between gut microbiota disturbance and the progression of renal dysfunction, it is hypothesized that administering probiotic bacteria to advanced CKD patients may have the benefit of delaying the deterioration of kidney function. Two animal studies provided evidence of probiotics attenuating renal fibrosis and improving renal function in mice with CKD. Zhu et al. [11] administered C57BL/6 mice with *L. casei* Zhang (a probiotic-producing bacterium that was isolated from Chinese fermented sour milk samples) or *L. acidophilus* for 4 weeks. Probiotic pretreatment resulted in lower serum BUN and creatinine with less pathological damage, such as necrosis, tubular dilatation, casts formation, and brush border loss [11]. Wang [34] et al. supplemented C57BL/6 mice with a high dose or low dose of probiotics containing *Lactobacillus acidophilus* (TYCA06), *Bifidobacterium longum* subspecies infantis (BLI-02), and *B. bifidum* (VDD088). Both low and high doses of probiotics significantly reduced the serum levels of BUN and creatinine. The inflammation in the renal cortex and glomerular corpuscles and normal compact renal tubules in the renal pelvis were all improved after treatment with probiotics, particularly in the high-dose group. In human studies, early research shows the effect of probiotic supplementation on CKD progression. The glomerular filtration rate was improved significantly in patients who were on the low-protein diet combined with prebiotics and probiotics [35]. Similar results were drawn from the other two clinical controlled trials on the probiotic intakes that were associated with a decline in the progression of CKD [13,36]. 

In addition, the metabolites of altered gut microbiota have been reported to be directly involved in the pathogenesis of cardiovascular disease and inflammation. A meta-analysis was performed on the effects of probiotics in CKD patients that supports the potential effect of probiotics supplementation in reducing levels of PCS [37], inflammation markers, and oxidative stress [38]. However, the meta-analysis in question reflected that the methodological quality varied across studies [38]. Several clinical trials have focused on the effects of probiotics, including dietary supplements, on inflammation in CKD, and yielded inconsistent results [8,20,23,29,32,39,40,41,42,43]. One trial from Mexico studied the different dose of lactobacillus casei Shirota (LcS) in CKD stages 3 and 4 and observed the higher dosages of LcS yielded better outcomes on decreasing the levels of inflammatory markers [23]. There was no evidence of a dose–effect relationship. Chen et al. studied the effects of the combinations of probiotic (Bifico) on interleukin 10 gene deficient (IL-10 KO) mice and Caco-2 cell monolayers [8]. IL-10 KO mice receiving the Bifico treatment had reduced the mucosal secretion of tumor necrosis factor-α and interferon-γ. The treatment of Caco-2 monolayers with Bifico or single-strain probiotics in vitro reduced the secretion of pro-inflammatory cytokines.

## 5. Effect of Probiotics in Dialysis Patients (Stage 5D)

With the deterioration of the residual kidney function, complications of ESKD and the dialysis procedure per se could lead to the dysbiosis of gut microbiota. Therefore, some studies assessed the probiotic treatment in either PD or HD patients. The constipation is reported to be common in patients on peritoneal dialysis (29%) and on hemodialysis (63%). The slowed transit time through the GI tract associated with constipation would lead to bacterial overgrowth in the stool that may contribute to the dysbiosis. Luo et al. [4] and Hu et al. [44] both compared the intestinal flora genome 16S rDNA sequencing in healthy people, CKD non-dialysis, HD patients, and PD patients in Chinese population. They concluded a remarkable difference in gut microbiota diversity before and after dialysis and inferred that PD and HD altered signal transduction and metabolic pathways. 

Table 2 depicted the randomized controlled trials of probiotic treatment in both HD and PD patients. Twelve studies were listed, with nine of them on HD and three on PD. The sample size ranged from 18 to 116 cases. Thirteen studies used probiotics alone and one study combined and probiotics. Most interventions lasted several weeks, and a few lasted as long as half a year [12,39].

### 5.1. HD

Many studies focused on the effect of probiotics and prebiotics in reducing uremic toxin with inconsistent results. Early studies have shown a distinct reduction in fecal p-cresol while plasma p-cresol was only slightly decreased in the hemodialysis population [53]. Another study found that probiotic consumption decreased the plasma concentration of IS (26) but only insignificantly decreased indoxyl glucuronide [12]. In a pilot study (n = 22 HD, uncontrolled), the investigators demonstrated a significant reduction in serum PCS, but not IS, when the prebiotic oligofructose-enriched inulin was taken [54]. In another study (n = 56 HD, placebo-controlled), Sirich et al. noted that resistant starch, a form of fiber that is resistant to digestion, significantly decreased serum IS, and possibly, also PCS [55]. Posen et al. found no effect of prebiotic arabinoxylan oligosaccharides (AXOS) on the serum levels of PCS, p-cresyl glucuronide (PCG), IS, and phenylacetylglutamine (PAG), and only a borderline significant effect (not adjusted for multi-comparison) on the serum levels of trimethylamine N-oxide (TMAO) [27]. Borges et al. drew the conclusion that, in the CKD population, probiotics failed to reduce uremic toxins and inflammatory markers [55]. Based on the above evidence, probiotic therapy should be chosen with caution in HD patients. Further studies should be performed on the use of probiotic therapy in patients on dialysis.

Cardiovascular disease is the most common cause for mortality in dialysis patients. During dialyses, only the free fraction of protein-bound solutes can be removed. Apart from traditional CV risk factors that are present in the vast majority of CKD patients, emerging evidence has suggested that non-traditional risk factors, such as oxidative stress and inflammation, may play a role in the pathogeneses of cardiovascular diseases [3]. Whether probiotics could reduce CVD risk remains elusive. The imbalance of intestinal microbiota and deleterious colonic microbial metabolism are closely associated with the production of microbiota-derived metabolites [56]. The majority of gut-derived uremic toxins have shown a direct role in mortality and complications under CKD. For example, IS and PCS are positively correlated with an increased CVD mortality in CKD patients [57]. Trimethylamine N-oxide (TMAO) was recognized as a pro-atherogenic metabolite that involved in developing CV disease in CKD patients [58]. Kaminski et al. observed that IS was independently associated with the markers of impaired endothelial function, oxidative stress, and monocyte activation determinants [59]. Taki et al. reported the oral administration of the *Bifidobacterium longum* in a gastroresistant seamless capsule significantly decreased the serum levels of homocysteine in HD patients [14]. There have been studies showing the relationship between gut-derived uremic toxins with inflammation as an important CVD risk factor. However, the causal effect of probiotics on reducing CVD mortality has not been confirmed by prospective randomized controlled trials.

### 5.2. PD

An alteration in the composition and function of the intestinal microbiome in PD patients has been reported [4,32]. Compared to non-dialysis patients or HD patients, PD patients have the continuous peritoneal dialysis fluid in the abdominal cavity affecting the physical and chemical environment of gastrointestinal tract, which may have an influence on intestinal flora and the effects of probiotics treatment. There have been few reports on the effects of prebiotic, probiotic, and/or synbiotic supplementation in PD patients [37,51,52]. It has been shown that oral probiotics could lower the serum levels of uric acid [52], endotoxin, and inflammatory cytokines [37], and improve the nutrition and quality of life [51]. All three of these studies suggested that probiotic supplementation in patients undergoing PD is safe and well tolerated. Some researchers administrated p-inulin (prebiotics) to PD patients [60,61] and revealed a preliminary association between the p-inulin treatment and the changes in microbiome, metabolic pathways, as well as the plasma metabolome in PD patients. No studies reported CVD events and infections as outcome measures. Overall, there is a substantial lack of evidence for the effects of probiotics on clinical outcomes including patient mortality in PD patients.

## 6. Limitations of the Studies Reviewed

There are limitations to the reviewed studies. Firstly, the sample size of the studies were usually not large and the follow-up times were not long enough. Secondly, the diversity of bacterial strains, dose, intervention period, and combined medications made it difficult to compare the studies. Thirdly, long-term prognosis and outcome, such as mortality, were not sufficiently focused upon in these studies. Additionally, the uremic milieu in the gut may not be favorable for the survival of probiotics limiting their potential health benefits.

## 7. Conclusions

To date, studies on the potential benefit of probiotics in CKD were generally performed among non-dialysis CKD patients. Most studies were performed with CKD patients and but less so in dialysis patients—especially peritoneal dialysis patients. Generally, the probiotic administration may have a potential benefit in improving symptoms and quality of life, reducing inflammation, and delaying the progression of kidney failure. Further research studies on using a larger sample size with longer follow-up duration and focus on clinical outcomes—including survival—are warranted to elucidate the significant clinical impact on the use of probiotics in CKD patients.

## Figures and Tables

**Table 1:** Summary of clinical trials on probiotic treatment used in patients with advanced CKD stage 3–5ND.

	Reference	Study Population	Country/Region	Type of Study	Sample Size	Endpoint Observed	Probiotic Type and Intervention Duration	Remarks
	Andreana De Mauri et al. [28]	eGFR < 25 mL/min/1.73 m^2^, non-dialysis	Italy	A single-centre, double-blind, placebo-controlled, randomized trial	60 patients	Uremic toxins, nutritional status, quality of life, the progression to end stage renal disease and dialysis initiation	a New formulation of probiotics (*Bifidobacterium longum* and *Lactobacillus reuteri*) 3 months	Probiotics to LPD may have an additional beneficial effect on the control and modulation of microbiota-derived and proatherogenic toxins in CKD patients
	I-Kuan Wang et al. [39]	CKD—Animal Model and CKD 3–5 patients	Taiwan	Animal studies and patients with stage 3–5 CKD and not on dialysis	C57BL/6 mice, 53 patients	In vitro indole assay for the probiotics treatment of CKD, clinical symptoms and pathological findings of mice with CKD; clinical outcomes of the human: the rate of decline of the eGFR, serum levels of endotoxin and proinflammatory cytokines, stool form and gastrointestinal symptoms	*Lactobacillus acidophilus* (TYCA06), *Bifidobacterium longum* subspecies infantis (BLI-02), and *B. bifidum* (VDD088) 6 months	A combination of probiotics might attenuate renal function deterioration in CKD mice and human patients
	Sandra Wagner et al. [20]	CKD patients with stage 3–5	France	Cross-sectional study	888 patients	Association between inflammation and the frequency of yoghurt/probiotic intake	Probiotics from yoghurts or dietary supplements 5 years	Consumption of yoghurts and probiotics is associated with a lower risk of inflammation in patients with CKD
	Catherine McFarlane et al. [43]	CKD patients with stage 3–4	Australia	A feasibility, double-blind, placebo-controlled, randomized trial	68 patients	Recruitment and retention rates as well as acceptability of the intervention	Synbiotic combined Bifidobacterium and Blautia spp for 12 months	Long-term synbiotic and probiotics supplementation was feasible and acceptable to patients with CKD, and it modified the gastrointestinal microbiome
	Carmela Cosola et al. [13]	Stage IIIb-IV CKD Patients	Italy	A randomized, single-blind, placebo-controlled, pilot trial	50 N = 23 CKD N = 27 healthy volunteers	Serum levels of microbiota-derived uremic toxins	Lactobacilli and Bifidobacteria species 2 months	The synbiotic NATUREN G ^®^ is effective in reducing serum free IS, small intestine permeability, abdominal pain and constipation syndromes in stage IIIb-IV CKD patients
	Mariadelina Simeoni et al. [32]	Stage 3a of CKD	Italy	An open-label, randomized, placebo-controlled study	28 patients	The impact of probiotic CKD administration protocol on fecal Lactobacillales and Bifidobacteria concentrations	Lactobacillales and Bifidobacteria 3 months	High-quality probiotics can effectively correct inflammatory indices, iron status and iPTH stabilization
	Paola Vanessa Miranda Alatriste et al. [23]	CKD stage 3 and stage 4	Mexico	A simple randomized, controlled clinical trial	30 patients	Change in the blood urea concentrations for patients treated with the 16 × 10 ^9^ dose lactobacillus casei shirota (LcS)	Lactobacillus casei shirota (LcS) 8 weeks	There was a >10% decrease in the serum urea concentrations with LcS in patients with stage 3 and 4 CRF
	B. Guida et al. [31]	CKD 3–4 stages	Italy	A double-blind, randomized placebo-controlled trial	30 patients	Total plasma p-cresol median concentra- tions and gastrointestinal symptoms	Synbiotic probinul-neutro 4 weeks	Probinul-neutro lowered total plasma p-cresol concentrations but did not ameliorate gastrointestinal symptoms in non-dialyzed CKD patients
	Amanda de Faria Barros et al. [42]	Non-dialysis CKD patients (stages 3–5)	Brazil	A randomized, double-blind, placebo-controlled trial	30 patients	Uremic toxins (cresyl sulfate, urea and TMAO) and inflammatory markers (IL-6 level and CRP)	*Streptococcus thermophilus*, *Lactobacillus acidophilus* and Bifidobacteria for 3 months	Probiotic supplementation did not result in expected benefits for non-dialysis CKD patients
	Natarajan Ranganathan et al. [12]	CKD stages 3 and 4	USA	A prospective, randomized, double-blind, placebo controlled crossover trial	46 patients	Biochemical parameters: blood urea nitrogen (BUN), serum creatinine, and uric acid and quality of life (QOL)	A mix of *L. acidophilus* KB27, *B. longum* KB31, and *S. thermophilus* KB19, for a total of 1.5 × 10 10 CFU 3 months	Supporting the use of the chosen probiotic formulation for bowel-based toxic solute extraction; QOL and BUN levels showed statistically significant differences in outcome between placebo and probiotic treatment
	Megan Rossi et al. [29]	CKD stages 4 and 5 not on dialysis	Australia	A randomized, double-blind, placebo-controlled, crossover trial	37 patients	p-cresyl sulfate (PCS) and indoxyl sulfate (IS); secondary outcomes include inflammatory markers and stool microbiota profile	Synbiotic therapy combined with Lactobacillus, Bifidobacteria, and Streptococcus 6 weeks	In patients with CKD, probiotics combined synbiotics did not significantly reduce serum IS but did decrease serum PCS and favorably modified the stool microbiome
Ruben Poesen et al. [27]		CKD 3b-4 stages	Belgium	A randomized, placebo-controlled, double-blind, cross-over study	40 patients	Primary outcome on serum levels of microbial metabolites and secondary outcome on 24 h urinary excretion of microbial metabolites and HOMA-IR	Prebiotic arabinoxylan oligosaccharides (AXOS) (10 g twice daily) and maltodextrin for 4 weeks	Could not demonstrate an influence of prebiotic AXOS on microbiota derived uremic retention solutes and insulin resistance in patients with CKD not yet on dialysis

**Table 2:** Summary of clinical trials on probiotics treatment used in patients with maintenance hemodialysis and peritoneal dialysis.

Reference	Study Population	Country/ Region	Type of Study	Sample Size	Endpoint Observed	Probiotic Type and Intervention Duration	Remarks
Chih-Yu Yang et al. [36]	Hemodialysis patients	Taiwan	A single-centre, double-blind, placebo-controlled, randomized trial	Animal model and 40 CKD patients and 22 healthy controls	The plasma levels of indoxyl sulfate and p-cresol sulfate in different groups, the relationship between gut microbiota, fecal indole content, and blood indoxyl sulfate level	Synbiotic and probiotics combination of *Lactobacillus* sp., *Bifidobacterium* sp., and *Streptococcus* sp. 5 weeks	Gut dysbiosis and renal function impairment could be ameliorated by synbiotic and probiotics treatment
Daniela Viramontes-Horner et al. [45]	Hemodialysis patients	Mexico	A double-blinded, placebo-controlled, randomized, clinical trial	22	Gastrointestinal symptoms (GISs) severity between intervention with control group	A mix of probiotics (*Lactobacillus acidophilus* NCFM and Bifidobacterium lactis Bi-07) 2 months	Administration of a symbiotic and probiotics gel is a safe and simple way to improve common GIS in dialysis patients
Farzad Eidi et al. [46]	Hemodialysis patients	Iran	A randomized controlled double-blind clinical trial	42	Uremic toxins between groups before and after Lactobacillus Rhamnosus use: total phenol and p-cresol	Lactobacillus Rhamnosus 4 weeks	Probiotics in hemodialysis patients can decrease serum phenolic uremic toxins.
Jose Cruz-Mora et al. [47]	Hemodialysis patients	Mexico	A randomized, double-blinded, placebo-controlled clinical trial	18	Bifidobacterial and lactobacilli counts, gastro intestinal symptoms scores in two groups	Probiotics (*Lactobacillus acidophilus* and *Bifidobacterium bifidum*) 2 months	Short-term symbiotic treatment in patients with ESRD can lead to the increase in Bifidobacterium counts, maintaining the intestinal microbial balance
Natalia A. Borges et al. [48]	Hemodialysis patients	Brazil	A randomized, double-blind, placebo-controlled study	46	Inflammatory markers (C-reactive protein and interleukin-6), uremic toxins plasma levels (indoxyl sulfate, p-cresyl sulfate, and indole-3-acetic acid), fecal pH, and gut microbiota profile	*Streptococcus thermophilus*, *Lactobacillus acidophilus* and *Bifidobacteria longum* 3 months	Probiotic supplementation failed to reduce uremic toxins and inflammatory markers
Alireza Soleimani et al. [49]	Hemodialysis patients	Iran	A parallel randomized double-blind placebo-controlled clinical trial	55	Fasting plasma glucose, serum insulin, homeostasis model of assessment-estimated insulin resistance, homeostasis model of assessment-estimated beta-cell function and HbA1c, and quantitative insulin sensitivity check index	Probiotics *Lactobacillus acidophilus*, *Lactobacillus casei* and *Bifidobacterium bifidum* 12 weeks	Probiotic supplementation for 12 weeks among diabetic hemodialysis patients had beneficial effects on parameters of glucose homeostasis, and some biomarkers of inflammation and oxidative stress
Ranganathan Natarajan et al. [12]	Hemodialysis patients	USA	A randomized, double-blind, placebo-controlled crossover study	22	Decline in WBC count and reductions in levels of C-reactive protein, and total indoxyl glucuronide, and QOL	Probiotic formulation—30 billion CFU of S; Thermophilus KB 19, *L. acidophilus* KB 27, and *B. longum* KB 31 6 months	Renadyl (strain-specific probiotic formulation) appeared to be safe to administer to ESRD patients on hemodialysis with stability in QOL assessment
Eunho Choi et al. [40]	Hemodialysis patients	Korea	A randomized, double-blind, placebo-controlled study	22	Various inflammatory parameters in hemodialysis (HD) patients	*Bifidobacterium bifidum* BGN4 and *Bifidobacterium longum* BORI 3 months	Probiotic supplementation reduced systemic inflammatory responses in HD patients with an increase in Tregs and a decrease in proinflammatory monocytes
Zahra Shariaty et al. [50]	Hemodialysis patients	Iran	A randomized parallel clinical trial	36	Hemoglobin (Hb) and serum C-reactive protein before and after intervention in probiotic and placebo groups	*Lactobacillus acidophilus*, Bifidobacterium and *Streptococcus thermophilus* 3 months	Probiotic supplementation decreased Hb fluctuations in hemodialysis patients but did not result in a significant increase in Hb levels.
Yangbin Pan et al. [51]	Peritoneal dialysis patients	China	A randomized controlled trial	116	High-sensitivity C-reactive protein and interleukin-6, serum albumin levels, upper arm circumference, and triceps skinfold thickness, scores on the SF-36 in different groups	*Bifidobacterium longum*, *Lactobacillus bulgaricus*, and *Streptococcus thermophilus* 2 months	Malnutrition and health-related quality of life partially improved after probiotic supplementation in patients undergoing PD
Shuiqing He et al. [52]	Peritoneal dialysis patients	China	A randomized, double-blind, placebo controlled crossover trial	16	Serum uric acid (UA) levels, fecal UA degradation capability, fecal metagenomic analysis to assess microbial composition and function	Inulin-type prebiotics was composed of a mixture of inulin and oligofructose 12 weeks	Inulin-type prebiotics can reduce serum UA levels in renal failure patients, and this urate-lowering effect could possibly be attributed to intestinal microbial degradation of UA
I.K. Wang et al. [34]	Peritoneal dialysis patients	Taiwan	A randomized, double-blind, placebo-controlled trial	39	The change in serum TNF-α, interferon gamma, IL-5, IL-6, IL-10, IL-17, and endotoxin levels before and six months after intervention	*Bifobacterium bifidum* A218, *Bifidobacterium catenulatum* A302, *Bifidobacterium longum* A101, and *Lactobacillus plantarum* A87 6 months	Probiotics could significantly reduce the serum levels of endotoxin, pro-inflammatory cytokines (TNF-α and IL-6), IL-5, increase the serum levels of anti-inflammatory cytokine (IL-10), and preserve residual renal function in PD patients.

## Data Availability

Not applicable.

## References

[1] Vaziri N.D., Wong J., Pahl M., Piceno Y.M., Yuan J., DeSantis T.Z., Ni Z., Nguyen T.-H., Andersen G.L. (2013). Chronic kidney disease alters intestinal microbial flora. Kidney Int..

[2] Mafra D., Lobo J.C., Barros A.F., Koppe L., Vaziri N.D., Fouque D. (2014). Role of altered intestinal microbiota in systemic inflammation and cardiovascular disease in chronic kidney disease. Future Microbiol..

[3] Szeto C.C., McIntyre C.W., Li P.K.-T. (2018). Circulating Bacterial Fragments as Cardiovascular Risk Factors in CKD. J. Am. Soc. Nephrol..

[4] Luo D., Zhao W., Lin Z., Wu J., Lin H., Li Y., Song J., Zhang J., Peng H. (2021). The Effects of Hemodialysis and Peritoneal Dialysis on the Gut Microbiota of End-Stage Renal Disease Patients, and the Relationship Between Gut Microbiota and Patient Prognoses. Front. Cell. Infect. Microbiol..

[5] Hill C., Guarner F., Reid G., Gibson G.R., Merenstein D.J., Pot B., Morelli L., Canani R.B., Flint H.J., Salminen S. (2014). Expert consensus document. The International Scientific Association for Probiotics and Prebiotics consensus statement on the scope and appropriate use of the term probiotic. Nat. Rev. Gastroenterol. Hepatol..

[6] Noda H., Akasaka N., Ohsugi M. (1994). Biotin production by bifidobacteria. J. Nutr. Sci. Vitaminol..

[7] Chen L., Zhang S., Wu S., Ren Z., Liu G., Wu J. (2021). Bacillus subtilis on Intestinal Epithelial Barrier Dysfunction in Caco-2 Cell Model and Mice Model of Lipopolysaccharide Stimulation. Front. Immunol..

[8] Shi C.-Z., Chen H.-Q., Liang Y., Xia Y., Yang Y.-Z., Yang J., Zhang J.-D., Wang S.-H., Liu J., Qin H.-L. (2014). Combined probiotic bacteria promotes intestinal epithelial barrier function in interleukin-10-gene-deficient mice. World J. Gastroenterol..

[9] Kailasapathy K., Chin J. (2000). Survival and therapeutic potential of probiotic organisms with reference to *Lactobacillus acidophilus* and Bifidobacterium spp.. Immunol. Cell Biol..

[10] Saarela M., Mogensen G., Fonden R., Matto J., Mattila-Sandholm T. (2000). Probiotic bacteria: Safety, functional and technological properties. J. Biotechnol..

[11] Zhu H., Cao C., Wu Z., Zhang Z., Wang M., Xu H., Zhao Z., Wang Y., Pei G., Yang Q. (2021). The probiotic *L. casei* Zhang slows the progression of acute and chronic kidney disease. Cell Metab..

[12] Natarajan R., Pechenyak B., Vyas U., Ranganathan P., Weinberg A., Liang P., Mallappallil M.C., Norin A.J., Friedman E.A., Saggi S. (2014). Randomized Controlled Trial of Strain-Specific Probiotic Formulation (Renadyl) in Dialysis Patients. Biomed Res. Int..

[13] Cosola C., Rocchetti M.T., di Bari I., Acquaviva P.M., Maranzano V., Corciulo S., di Ciaula A., di Palo D.M., la Forgia F.M., Fontana S. (2021). An Innovative Synbiotic Formulation Decreases Free Serum Indoxyl Sulfate, Small Intestine Permeability and Ameliorates Gastrointestinal Symptoms in a Randomized Pilot Trial in Stage IIIb-IV CKD Patients. Toxins.

[14] Taki K., Takayama F., Niwa T. (2005). Beneficial effects of Bifidobacteria in a gastroresistant seamless capsule on hyperhomocysteinemia in hemodialysis patients. J. Ren. Nutr..

[15] Haring B., Selvin E., Liang M., Coresh J., Grams M.E., Natalia P.I., Steffen L.M., Rebholz C.M. (2017). Dietary Protein Sources and Risk for Incident Chronic Kidney Disease: Results from the Atherosclerosis Risk in Communities (ARIC) Study. J. Ren. Nutr..

[16] Kelly J.T., Palmer S.C., Wai S.N., Ruospo M., Carrero J.-J., Campbell K.L., Strippoli G.F.M. (2017). Healthy Dietary Patterns and Risk of Mortality and ESRD in CKD: A Meta-Analysis of Cohort Studies. Clin. J. Am. Soc. Nephrol..

[17] Lew Q.J., Jafar T.H., Koh H.W., Jin A., Chow K.Y., Yuan J.M., Koh W.P. (2017). Red Meat Intake and Risk of ESRD. J. Am. Soc. Nephrol..

[18] De Preter V., Vanhoutte T., Huys G., Swings J., De-Vuyst L., Rutgeerts P., Verbeke K. (2007). Effects of Lactobacillus casei Shirota, Bifidobacterium breve, and oligofructose-enriched inulin on colonic nitrogen-protein metabolism in healthy humans. Am. J. Physiol. -Gastrointest. Liver Physiol..

[19] Patel K.P., Luo F.J., Plummer N.S., Hostetter T.H., Meyer T.W. (2012). The production of p-cresol sulfate and indoxyl sulfate in vegetarians versus omnivores. Clin. J. Am. Soc. Nephrol..

[20] Wagner S., Merkling T., Metzger M., Koppe L., Laville M., Boutron-Ruault M.C., Frimat L., Combe C., Massy Z.A., Stengel B. (2022). Probiotic Intake and Inflammation in Patients with Chronic Kidney Disease: An Analysis of the CKD-REIN Cohort. Front. Nutr..

[21] Armani R.G., Ramezani A., Yasir A., Sharama S., Canziani M.E.F., Raj D.S. (2017). Gut Microbiome in Chronic Kidney Disease. Curr. Hypertens Rep..

[22] De Angelis M., Montemurno E., Piccolo M., Vannini L., Lauriero G., Maranzano V., Gozzi G., Serrazanetti D., Dalfino G., Gobbetti M. (2014). Microbiota and metabolome associated with immunoglobulin A nephropathy (IgAN). PLoS ONE.

[23] Miranda A.P., Urbina A.R., Gomez E.C., Espinosa C.M.L. (2014). Effect of probiotics on human blood urea levels in patients with chronic renal failure. Nutr. Hosp..

[24] Jiang S., Xie S., Lv D., Zhang Y., Deng J., Zeng L., Chen Y. (2016). A reduction in the butyrate producing species Roseburia spp. and Faecalibacterium prausnitzii is associated with chronic kidney disease progression. Antonie Van Leeuwenhoek.

[25] Miraghajani M., Zaghian N., Mirlohi M., Awat F., Reza G. (2017). The Impact of Probiotic Soy Milk Consumption on Oxidative Stress Among Type 2 Diabetic Kidney Disease Patients: A Randomized Controlled Clinical Trial. J. Ren. Nutr..

[26] Rusu I.G., Suharoschi R., Vodnar D.C., Pop C.R., Socaci S.A., Vulturar R., Istrati M., Moroșan I., Fărcaș A.C., Kerezsi A.D. (2020). Iron Supplementation Influence on the Gut Microbiota and Probiotic Intake Effect in Iron Deficiency—A Literature-Based Review. Nutrients.

[27] Poesen R., Evenepoel P., de Loor H., Delcour J.A., Courtin C.M., Kuypers D., Augustijns P., Verbeke K., Meijers B. (2016). The Influence of Prebiotic Arabinoxylan Oligosaccharides on Microbiota Derived Uremic Retention Solutes in Patients with Chronic Kidney Disease: A Randomized Controlled Trial. PLoS ONE.

[28] De Mauri A., Carrera D., Bagnati M., Rolla R., Vidali M., Chiarinotti D., Pane M., Amoruso A., del Piano M. (2022). Probiotics-Supplemented Low-Protein Diet for Microbiota Modulation in Patients with Advanced Chronic Kidney Disease (ProLowCKD): Results from a Placebo-Controlled Randomized Trial. Nutrients.

[29] Rossi M., Johnson D.W., Morrison M., Pascoe E.M., Coombes J.S., Forbes J.M., Szeto C.-C., McWhinney B.C., Ungerer J.P.J., Campbell K.L. (2016). Synbiotics Easing Renal Failure by Improving Gut Microbiology (SYNERGY): A Randomized Trial. Clin. J. Am. Soc. Nephrol..

[30] Ranganathan N., Ranganathan P., Friedman E.A., Joseph A., Delano B., Goldfarb D.S., Tam P., Rao A.V., Anteyi E., Musso C.G. (2010). Pilot study of probiotic dietary supplementation for promoting healthy kidney function in patients with chronic kidney disease. Adv. Ther..

[31] Guida B., Germano R., Trio R., Russo D., Memoli B., Grumetto L., Barbato F., Cataldi M. (2014). Effect of short-term synbiotic treatment on plasma p-cresol levels in patients with chronic renal failure: A randomized clinical trial. Nutr. Metab. Cardiovasc. Dis..

[32] Simeoni M., Citraro M.L., Cerantonio A., Deodato F., Provenzano M., Cianfrone P., Capria M., Corrado S., Libri E., Comi A. (2019). An open-label, randomized, placebo-controlled study on the effectiveness of a novel probiotics administration protocol (ProbiotiCKD) in patients with mild renal insufficiency (stage 3a of CKD). Eur. J. Nutr..

[33] Tayebi-Khosroshahi H., Habibzadeh A., Niknafs B., Ghotaslou R., Yeganeh S.F., Ghojazadeh M., Moghaddaszadeh M., Parkhide S. (2016). The effect of lactulose supplementation on fecal microflora of patients with chronic kidney disease; a randomized clinical trial. J. Ren. Inj. Prev..

[34] Wang I.-K., Yen T.-H., Hsieh P.-S., Ho H.-H., Kuo Y.-W., Huang Y.-Y., Kuo Y.-L., Li C.-Y., Lin H.-C., Wang J.-Y. (2021). Effect of a Probiotic Combination in an Experimental Mouse Model and Clinical Patients with Chronic Kidney Disease: A Pilot Study. Front. Nutr..

[35] Pavan M. (2016). Influence of prebiotic and probiotic supplementation on the progression of chronic kidney disease. Minerva Urol. E Nefrol..

[36] Yang C.-Y., Chen T.-W., Lu W.-L., Liang S.-S., Huang H.-D., Tseng C.-P., Tarng D.-C. (2021). Synbiotics Alleviate the Gut Indole Load and Dysbiosis in Chronic Kidney Disease. Cells.

[37] Jia L., Jia Q., Yang J., Jia R., Zhang H. (2018). Efficacy of Probiotics Supplementation on Chronic Kidney Disease: A Systematic Review and Meta-Analysis. Kidney Blood Press Res..

[38] Zheng H.J., Guo J., Wang Q., Wang L., Wang Y., Zhang F., Huang W.-J., Zhang W., Liu W.J., Wang Y. (2021). Probiotics, prebiotics, and synbiotics for the improvement of metabolic profiles in patients with chronic kidney disease: A systematic review and meta-analysis of randomized controlled trials. Crit. Rev. Food Sci. Nutr..

[39] Wang I.K., Wu Y.Y., Yang Y.F., Ting I.W., Lin C.C., Yen T.H., Chen J.-H., Wang C.-H., Huang C.-C., Lin H.-C. (2015). The effect of probiotics on serum levels of cytokine and endotoxin in peritoneal dialysis patients: A randomised, double-blind, placebo-controlled trial. Benef. Microbes.

[40] Choi E., Yang J., Ji G., Park M.S., Seong Y., Oh S.W., Kim M.G., Cho W.Y., Jo S.K. (2022). The effect of probiotic supplementation on systemic inflammation in dialysis patients. Kidney Res. Clin. Pract..

[41] Wang P., Peng Y., Guo Y., Zhao Y. (2022). The efficacy of probiotic preparations on inflammatory cytokines in patients with chronic kidney disease. Medicine.

[42] de Faria Barros A., Borges N.A., Nakao L.S. (2018). Effects of probiotic supplementation on inflammatory biomarkers and uremic toxins in non-dialysis chronic kidney patients: A double-blind, randomized, placebo-controlled trial. J. Funct. Foods.

[43] McFarlane C., Krishnasamy R., Stanton T., Savill E., Snelson M., Mihala G., Kelly J.T., Morrison M., Johnson D.W., Campbell K.L. (2021). Synbiotics Easing Renal Failure by Improving Gut Microbiology II (SYNERGY II): A Feasibility Randomized Controlled Trial. Nutrients.

[44] Hu J., Zhong X., Yan J., Zhou D., Qin D., Xiao X., Zheng Y., Liu Y. (2020). High-throughput sequencing analysis of intestinal flora changes in ESRD and CKD patients. BMC Nephrol..

[45] Viramontes-Hörner D., Márquez-Sandoval F., Martín-del-Campo F., Vizmanos-Lamotte B., Sandoval-Rodriguez A., Armendariz-Borunda J., García-Bejarano H., Renoirte-López K., García-García G. (2015). Effect of a Symbiotic Gel (*Lactobacillus acidophilus* + Bifidobacterium lactis + Inulin) on Presence and Severity of Gastrointestinal Symptoms in Hemodialysis Patients. J. Ren. Nutr..

[46] Eidi F., Poor Reza Gholi F., Ostadrahimi A., Dalili N., Samadian F., Barzegari A. (2018). Effect of Lactobacillus Rhamnosus on serum uremic toxins (phenol and P-Cresol) in hemodialysis patients: A double blind randomized clinical trial. Clin. Nutr. ESPEN.

[47] Cruz-Mora J., Martinez-Hernandez N.E., Martin D.C.F., Viramontes-Horner D., Vizmanos-Lamotte B., Munoz-Valle J.F., García-García G., Parra-Rojas I., Castro-Alarcón N. (2014). Effects of a symbiotic on gut microbiota in Mexican patients with end-stage renal disease. J. Ren. Nutr..

[48] Borges N.A., Carmo F.L., Stockler-Pinto M.B., de Brito J.S., Dolenga C.J., Ferreira D.C., Nakao L.S., Rosado A., Fouque D., Mafra D. (2018). Probiotic Supplementation in Chronic Kidney Disease: A Double-blind, Randomized, Placebo-controlled Trial. J. Ren. Nutr..

[49] Soleimani A., Zarrati M.M., Bahmani F., Taghizadeh M., Ramezani M., Tajabadi-Ebrahimi M., Jafari P., Esmaillzadeh A., Asemi Z. (2017). Probiotic supplementation in diabetic hemodialysis patients has beneficial metabolic effects. Kidney Int..

[50] Shariaty Z., Mahmoodi Shan G.M., Farajollahi M., Amerian M., Pour N. (2017). The effects of probiotic supplement on hemoglobin in chronic renal failure patients under hemodialysis: A randomized clinical trial. J. Res. Med. Sci..

[51] Pan Y., Yang L., Dai B., Lin B., Lin S., Lin E. (2021). Effects of Probiotics on Malnutrition and Health-Related Quality of Life in Patients Undergoing Peritoneal Dialysis: A Randomized Controlled Trial. J. Ren. Nutr..

[52] He S., Xiong Q., Tian C., Li L., Zhao J., Lin X., Guo X., He Y., Liang W., Zuo X. (2022). Inulin-type prebiotics reduce serum uric acid levels via gut microbiota modulation: A randomized, controlled crossover trial in peritoneal dialysis patients. Eur. J. Nutr..

[53] Hida M., Aiba Y., Sawamura S., Suzuki N., Satoh T., Koga Y. (1996). Inhibition of the accumulation of uremic toxins in the blood and their precursors in the feces after oral administration of Lebenin, a lactic acid bacteria preparation, to uremic patients undergoing hemodialysis. Nephron.

[54] Meijers B.K., De Preter V., Verbeke K., Vanrenterghem Y., Evenepoel P. (2010). P-Cresyl sulfate serum concentrations in haemodialysis patients are reduced by the prebiotic oligofructose-enriched inulin. Nephrol. Dial. Transplant..

[55] Sirich T.L., Plummer N.S., Gardner C.D., Hostetter T.H., Meyer T.W. (2014). Effect of increasing dietary fiber on plasma levels of colon-derived solutes in hemodialysis patients. Clin. J. Am. Soc. Nephrol..

[56] Barrios C., Beaumont M., Pallister T., Villar J., Goodrich J.K., Clark A., Pascual J., Ley R.E., Spector T.D., Bell J.T. (2015). Gut-Microbiota-Metabolite Axis in Early Renal Function Decline. PLoS ONE.

[57] Tang W.H., Wang Z., Levison B.S., Koeth R.A., Britt E.B., Fu X., Fu X., Wu Y., Hazen S.L. (2013). Intestinal microbial metabolism of phosphatidylcholine and cardiovascular risk. New Engl. J. Med..

[58] Tang W.H., Wang Z., Kennedy D.J., Wu Y., Buffa J.A., Agatisa-Boyle B., Li X.S., Levison B.S., Hazen S.L. (2015). Gut microbiota-dependent trimethylamine N-oxide (TMAO) pathway contributes to both development of renal insufficiency and mortality risk in chronic kidney disease. Circ. Res..

[59] Kaminski T.W., Pawlak K., Karbowska M., Mysliwiec M., Pawlak D. (2017). Indoxyl sulfate-the uremic toxin linking hemostatic system disturbances with the prevalence of cardiovascular disease in patients with chronic kidney disease. BMC Nephrol..

[60] Li L., Xiong Q., Zhao J., Lin X., He S., Wu N., Yao Y., Liang W., Zuo X., Ying C. (2020). Inulin-type fructan intervention restricts the increase in gut microbiome–generated indole in patients with peritoneal dialysis: A randomized crossover study. Am. J. Clin. Nutr..

[61] Gao B., Alonzo-Palma N., Brooks B., Jose A., Barupal D., Jagadeesan M., Nobakht E., Collins A., Ramezani A., Omar B. (2020). A Pilot Study on the Effect of Prebiotic on Host-Microbial Co-metabolism in Peritoneal Dialysis Patients. Kidney Int. Rep..

